# Combating PDAC Drug Resistance: The Role of Ref-1 Inhibitors in Accelerating Progress in Pancreatic Cancer Research

**DOI:** 10.33696/signaling.5.126

**Published:** 2024

**Authors:** Eyram K. Kpenu, Mark R. Kelley

**Affiliations:** 1 Department of Pediatrics and Herman B Wells Center for Pediatric Research, Indiana University School of Medicine, Indianapolis, IN, USA; 2 Indiana University Simon Comprehensive Cancer Center, Indiana University School of Medicine, Indianapolis, IN, USA; 3 Indiana University School of Medicine, Department of Biochemistry and Molecular Biology, Indianapolis, IN, USA

**Keywords:** APE1/Ref-1, Redox regulation, PDAC, Drug development, PDAC model, Redox inhibition, Transcription factor

## Abstract

Pancreatic Ductal Adenocarcinoma (PDAC) remains one of the most lethal solid tumor diagnoses given its limited treatment options and dismal prognosis. Its complex tumor microenvironment (TME), heterogeneity, and high propensity for drug resistance are major obstacles in developing effective therapies.

Here, we highlight the critical role of Redox effector 1 (Ref-1) in PDAC progression and drug resistance, focusing on its redox regulation of key transcription factors (TFs) such as STAT3, HIF1α, and NF-κB, which are pivotal for tumor survival, proliferation, and immune evasion. We discuss the development of novel Ref-1 inhibitors, including second-generation compounds with enhanced potency and improved pharmacokinetic profiles, which have shown significant promise in preclinical models. These inhibitors disrupt Ref-1’s redox function, leading to decreased TF activity and increased chemosensitivity in PDAC cells. We further detail our utilization of advanced preclinical models, such as 3D spheroids, organoids, and Tumor-Microenvironment-on-Chip (T-MOC) systems, which better simulate the complex conditions of the PDAC TME and improve the predictive power of therapeutic responses.

By targeting Ref-1 and its associated pathways, in conjunction with improved models, more replicative of PDAC’s TME, we are focused on approaches which hold the potential to overcome current therapeutic limitations and advance the development of more effective treatments for PDAC. Our findings suggest that integrating Ref-1 inhibitors into combination therapies could disrupt multiple survival mechanisms within the tumor, offering new hope for improving outcomes in this challenging cancer.

## Introduction

Pancreatic ductal adenocarcinoma (PDAC) is among the deadliest solid tumor cancer diagnoses. Its incidence continues to rise and, with current projections, will be the second leading cause of cancer death in the United States by 2030 [1,2]. Surgical resection is the only curative course for PDAC; however, this is an often-unavailable option for most patients as the disease typically presents with non-specific symptoms in its initial stages and is thus not identified and diagnosed until well after it has already metastasized.

PDAC treatment currently consists of first-line therapy with FOLFIRINOX, which is a combination of 5-fluorouracil (5-FU), leucovorin, irinotecan, and oxaliplatin. There is also Gemcitabine paired with Abraxane (nab-paclitaxel) [3], and chemoradiation is increasingly being used for patients with borderline resectable tumors to shrink the tumor and increase the chances of successful surgical resection [4]. While these options represent an acceleration of progress in the field, the prognosis for PDAC remains poor, and the five-year survival for non-surgically resected patients has only reached 12% [5].

This unfortunate reality is in-part due to the difficulties with PDAC drug development which is mired with challenges. The usually late detection of the cancer along with the genetic heterogeneity of PDAC tumors, both between patients and within individual tumors, complicates the development of universally effective targeted therapies [6]. The dense stromal tissue characteristic of the PDAC tumor microenvironment (TME) creates a physical barrier that impedes drug delivery, while also promoting an immunosuppressive environment. PDAC tumors exhibit significant metabolic adaptations which support their growth under nutrient-limited conditions and contribute to chemoresistance [5]. The high rate of inherent and acquired resistance to chemotherapeutic agents further complicates treatment. These factors combined have resulted in high clinical trials attrition with PDAC possessing one of the highest rates of failure in phase 3 clinical trials [5,7]. Thus, there is a great need for novel therapeutic options coupled with more rigorous preclinical models which can better translate these therapies to clinical effectiveness.

### PDAC, the TME, and Drug Development Challenges

#### The PDAC tumor microenvironment and ECM barriers

Crucial to PDAC homeostasis, disease progression, and chemoresistance is its tumor microenvironment (TME). The TME refers to the complex architecture surrounding the pancreatic tumor cells. It arises as a desmoplastic tissue response which stems from crosstalk between tumor cells and the surrounding stroma, and is further driven by genetic mutations, notably in the KRAS oncogene but also including other factors such as the tumor suppressor genes TP53 and SMAD4, as well as the cell cycle regulator CDKN2A [8–10]. This desmoplastic stroma enriched with extracellular matrix (ECM) components such as collagen and hyaluronan, forms a physical barrier around the tumor. Enmeshed within this complex stroma, are several different cell types, including pancreatic stellate cells (PSCs), immune cells, and cancer-associated fibroblasts (CAFs).

CAFs are the most abundant cell type in the PDAC TME, and they principally function to secrete the components of the ECM. The ECM not only acts as a physical barrier but plays an active role in the signaling and metabolic interactions within the TME [11]. Collagen, the most abundant ECM protein, can influence immune cell infiltration and tumor cell behavior through signaling pathways like the discoidin domain receptor signaling [12]. Other proteins function in similar cancer promoting roles. For instance, hyaluronan increases interstitial pressure through water retention, a process that, over time, can collapse blood vessels and hinder drug delivery [13].

#### PDAC immune suppression and drug resistance

Crosstalk between the tumor cells, CAFs, and immune cells leads to alterations in the immune cell subtypes, and this rewiring produces an immunosuppressive environment characterized by the presence of suppressive immune cells like regulatory T cells (Tregs), myeloid-derived suppressor cells (MDSCs), and tumor-associated macrophages (TAMs) [11]. Moreover, unlike more immunogenic tumors like melanoma or lung cancer, PDAC exhibits a very low tumor mutational burden, resulting in a limited number of neoantigens that can be recognized by the immune system. This leads to poor immune recognition which is exacerbated by the tendency of PDAC tumors to downregulate or sequester MHC class I molecules, thus preventing the effective immune targeting of cancer cells. This complex interplay of physical and biochemical barriers within the TME, has rendered checkpoint inhibitors and other immunotherapeutic strategies largely ineffective to date [14].

The TME plays a pivotal role in promoting drug resistance through a combination of cellular and biochemical mechanisms. Cancer stem cells (CSCs) within the TME are known to resist chemotherapy by utilizing efficient DNA repair mechanisms and overexpressing drug-efflux transporters, which expel therapeutic agents from the cell [15]. Additionally, the TME fosters epithelial-mesenchymal transition (EMT), a process that not only contributes to increased tumor cell invasiveness but also endows tumor cells with mesenchymal characteristics that are highly resistant to drugs. This transition often results in the formation of CSCs which can further withstand standard treatments. This is worsened by hypoxia within the TME which exacerbates this resistance by activating survival pathways and reducing cell sensitivity to apoptosis [16].

The TME significantly contributes to the difficulties in tackling PDAC. Collectively, its effects create a fortified environment that shields PDAC cells from the effects of chemotherapy and renders traditional treatment approaches less effective. Effective modeling of the TME including its ECM components, fibroblast activity, and altered immune cell population remains critical for advancing PDAC therapy and overcoming the critical failures seen in the translation of preclinical to clinical studies.

### Ref-1 in PDAC Homeostasis

#### Ref-1/APE1: Redox signaling and DNA repair

Redox effector 1 (Ref-1) also known as Apurinic/apyrimidinic endonuclease 1 (APE1), hereafter simply Ref-1, is a multifunctional protein which facilitates multiple cellular functions including DNA repair, redox signaling, and miRNA processing [17,18]. In DNA repair, Ref-1 is an endonuclease in the base excision repair (BER) pathway, repairing oxidative and alkylating damage in DNA by cleaving the phosphodiester backbone at abasic sites thus facilitating the replacement of damaged bases. In its redox signaling role, Ref-1 regulates the redox state of a number of transcription factors (TFs) including HIF-1α, AP-1, STAT3, and NF-κB. Ref-1 modulates the redox state of specific cysteine (Cys) residues within these TFs by facilitating the reversible oxidation and reduction of thiol groups (-SH) on these residues, activating them and enhancing their DNA binding capacity. By maintaining these cysteine residues in their reduced, active form, Ref-1 promotes the DNA-binding activity of its target TFs and thus regulates their role in gene expression [17,19]. Here we will focus chiefly on Ref-1’s redox signaling capabilities.

#### Role of Ref-1 in regulating transcription factors

**Figure 1.**
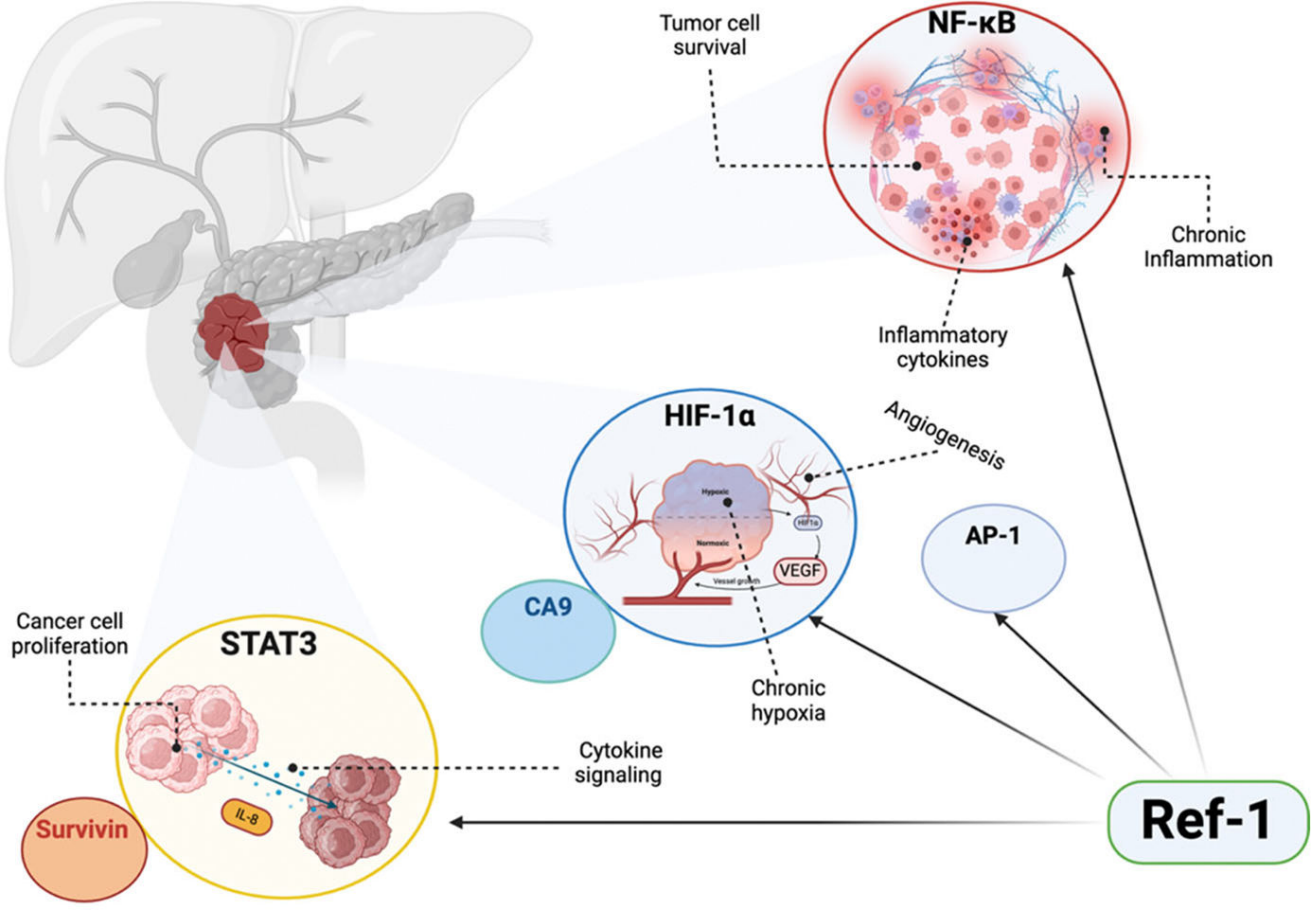
Ref-1 mediated regulation of key transcription factors driving PDAC progression and drug resistance. Ref-1 regulates multiple transcription factors that contribute to PDAC progression and resistance mechanisms. This schematic illustrates the roles of NF-κB, STAT3, and HIF-1α in the PDAC tumor microenvironment. Ref-1 activates NF-κB, promoting tumor cell survival and chronic inflammation through the release of inflammatory cytokines. Additionally, Ref-1 regulates STAT3, which drives cancer cell proliferation, cytokine signaling, and upregulates the anti-apoptotic protein survivin. Under hypoxic conditions, Ref-1 modulates HIF-1α, which promotes angiogenesis, contributes to chronic hypoxia, and activates CA9 which aids in maintaining pH balance under hypoxic conditions [19]. Ref-1’s regulation of these pathways underscores its central role in orchestrating transcriptional responses that enhance PDAC survival, proliferation, and resistance to therapy.

Ref-1 overexpression has been detailed in multiple cancer types, notably in PDAC but also colon, ovarian, bladder, sarcomas, and lung [19]. It is a negative prognostic factor as Ref-1 overexpression is associated with enhanced tumor growth, survival, and drug resistance [20]. In PDAC, Ref-1 is a significant driver of tumor progression through the regulation of critical transcription factor and metabolic pathways (Figure 1). By amplifying the activity of its target TFs, Ref-1 enhances their downstream signaling, ultimately resulting in increased tumor cell proliferation, survival, invasion, and metastasis. Additionally, Ref-1 activity alters metabolic pathways, including oxidative phosphorylation (OXPHOS) and the tricarboxylic acid (TCA) cycle, which are crucial for tumor growth and survival under hypoxic conditions [20,21].

### Ref-1 as a PDAC Target

#### Dual targeting of Ref-1 and STAT3

We have previously reported using the selective Ref-1 inhibitor APX3330 to effectively target Ref-1’s redox function within PDAC cell lines [19,22]. As we have demonstrated, targeting Ref-1’s activation of its TF targets offers a promising therapeutic strategy to disrupt PDAC tumor survival pathways and enhance chemosensitivity [17,22]. We have further detailed the results of simultaneously targeting Ref-1 in conjunction with signal transducer and activator of transcription 3 (STAT3), a TF under Ref-1 redox regulation. STAT3 is mediator of cytokine signaling within the PDAC TME, and in this capacity, it promotes PDAC tumor cell survival, immune evasion, and proliferation [23–25]. Ref-1’s reduction of key STAT3 cysteine residues is essential for its DNA binding ability which is necessary for the activation of its target genes [26]. Pharmacological inhibition of Ref-1’s redox function using APX3330 significantly reduces STAT3 DNA binding and transcriptional activity ultimately producing a dose-dependent reduction in STAT3 target genes such as Survivin. The dual targeting of Ref-1 and STAT3 leads to a substantial decrease in the proliferation and survival of PDAC cells [26]. This combined inhibition induces marked apoptosis, and effectively reduces PDAC cell migration significantly more than targeting either protein alone. Knockdown of Ref-1 does not affect the overall levels of STAT3 protein or its phosphorylation status, indicating that Ref-1’s regulatory role is specific to its redox function on STAT3 DNA binding.

#### Inhibiting HIF1α and Ref-1 under hypoxia

The success with targeting STAT3 is paralleled with hypoxiainducible factor 1 Alpha (HIF1α), a transcription factor that responds to hypoxic conditions in the cellular environment and drives survival by the activation of genes that promote angiogenesis and metabolic adaptations [27,28]. Ref-1 enhances the activity of HIF1α under hypoxic conditions by maintaining its reduced state enabling DNA binding and increasing transcriptional activity. Carbonic Anhydrase IX (CA9), an enzyme regulated by HIF1α, facilitates the maintenance of pH balance in hypoxic tumor cells, thereby enhancing cell survival. Inhibition of Ref-1 with APX3330 leads to decreased HIF1α activity and, consequently, a reduction in the expression of CA9 [29].

Combined targeting of Ref-1 and CA9 with APX3330 and the CA9 inhibitor SLC-0111 leads to significant reduction in PDAC cell viability and growth under hypoxic conditions [29]. Dual targeting led to a more pronounced acidification of the intracellular environment and greater inhibition of cell proliferation and survival than targeting either protein alone. The combined Ref-1 and CA9 inhibition produced enhanced tumor cell killing even in the protective environment of the tumor stroma. Notably, these studies utilized patient-derived 3D co-culture models, which included tumor cells and CAFs thus demonstrating the effectiveness of the dual-targeting in a more physiologically relevant setting [29]. The 3D models revealed that dual inhibition disrupts tumor-stroma interactions. Thus, this dual-targeting strategy not only impairs tumor cell adaptation to hypoxia but also disrupts the protective TME indicating a potential therapeutic strategy for overcoming PDAC resistance to conventional treatments.

#### Combating inflammation: Ref-1 and NF-κB

Another Ref-1 TF target is RelA (p65), a subunit of NF-κB, a transcription factor known principally for its promotion of inflammation. In PDAC, NF-κB is constitutively active, driving the expression of pro-inflammatory cytokines and survival genes, contributing to chemoresistance [30]. Ref-1 maintains RelA in an active state, promoting the transcription of genes involved in inflammation and tumor cell survival. This interaction is crucial for sustaining the chronic inflammatory environment in PDAC. Inhibiting Ref-1 activity with APX3330 reduces RelA activity, decreasing inflammation and enhancing tumor cell sensitivity to chemotherapy [31]. PRDX1, a known redox modulator of Ref-1, was found to significantly influence the cellular response to Ref-1 inhibition. Knockdown of PRDX1 in RelA-proficient cells dramatically increased sensitivity to Ref-1 inhibitors, highlighting the role of PRDX1 in modulating redox homeostasis and RelA activity.

#### ISR pathway and Ref-1 inhibition

In addition to targeting specific TF pathways, we have previously reported on the potential of combining Ref-1 inhibition with modulation of the integrated stress response (ISR) pathway in PDAC [32]. The ISR is a crucial cellular defense mechanism wherein cells adapt to various stress conditions by modulating protein synthesis and activating stress-related genes [33]. The ISR primarily functions through the phosphorylation of the eukaryotic initiation factor 2 alpha (eIF2α), which leads to a reduction in global protein synthesis while selectively enhancing the translation of stress-responsive genes such as ATF4 [34].

Ref-1 knockdown activates the ISR pathway, and inhibition of Ref-1 with the second-generation redox inhibitors APX2009 and APX2014 produces a dose dependent activation of the ISR pathway through the PERK-eIF2α-ATF4 axis as evidenced by increased phosphorylation of eIF2α and PERK and elevated ATF4 levels [32,35]. This suggests that the cellular stress induced by Ref-1 inhibition triggers the ISR as a compensatory mechanism and may be one of the mechanisms underlying Ref-1 cytotoxicity. The simultaneous inhibition of Ref-1, with either APX2009 or APX2014, and PERK, with the selective inhibitor AMG-44, led to a marked increase in cell death compared to either treatment alone. Notably, while the effects of PERK inhibition were minimal on both tumor and CAF lines in monolayer, they were particularly evident in 3D co-culture models, with the combined treatment more effective in killing PDAC cells even in the presence of protective stromal CAFs [32]. These results not only demonstrated the efficacy of dual inhibition of Ref-1 and the ISR pathway, but they highlight the significance of utilizing 3D models which better capture the complexity of PDAC tumors.

Therapeutic implications of our work suggest that combining Ref-1 inhibitors with specific TF inhibitors can enhance therapeutic efficacy by simultaneously disrupting both the redox activation and the downstream signaling pathways of these TFs in PDAC. By blocking TF activation through redox inhibition alongside the targeting of associated signaling pathways, this combination approach can effectively disrupt multiple survival mechanisms within the tumor, essentially a synthetic lethal approach to tumor killing. Further, incorporating Ref-1 inhibitors into existing chemotherapy regimens could enhance tumor cell sensitivity to treatment, thereby enhancing overall treatment efficacy. Utilizing Ref-1 inhibitors in combination with agents targeting the TME (e.g., anti-angiogenic drugs, immunotherapies) and the ISR may disrupt tumor-stroma interactions and improve therapeutic outcomes.

#### Second-generation Ref-1 Inhibitors

In a recent paper, we detailed the development of new Ref1 inhibitors which demonstrate improved efficacy over our parent compound APX3330 [36]. The research detailed the development of the new Ref-1 inhibitors: APX2009, APX2014, APX2044, APX2051, and APX2053 (Table 1). These compounds were developed through a structure-activity relationship (SAR) approach, resulting in inhibitors with 5–10 times greater potency than APX3330. These new inhibitors exhibit better pharmacokinetics (PK), metabolic stability, and safety profiles, making them more suitable for clinical applications.

The new inhibitors were evaluated with an array of techniques. *In silico* ADMET (absorption, distribution, metabolism, excretion-toxicity) properties were evaluated. PK studies demonstrated that the new compounds had better stability and bioavailability compared to APX3330. The compounds exhibited significantly higher plasma stability and lower *in vivo* clearance, indicating a better oral bioavailability profile [36]. NMR spectroscopy confirmed direct interactions between the new inhibitors and Ref-1 protein, indicating effective target engagement. Transcription factor activity assays revealed that the new inhibitors effectively blocked the activity of NF-κB, HIF1α, and STAT3 in PDAC tumor cells, with dose-dependent reductions in transcription factor activity observed. Mitochondrial function assays demonstrated that the inhibitors reduced mitochondrial function and NADPH levels, indicating a shift towards a more oxidized cellular environment. Proteomics analysis revealed significant changes in protein expression associated with cell cycle, DNA repair, mitochondrial function, and signaling pathways following Ref-1 inhibition [36].

We have previously described the critical role of Cysteine 65 (Cys65) in the redox function of Ref-1. Cys65 functions as the primary nucleophile in the thiol-disulfide exchange reaction that facilitates Ref-1’s reduction capability [37]. Mutating Cys65 in Ref-1 significantly impaired its redox regulation of critical TFs such as HIF-1α and STAT3. In mutant cell lines where Cys65 was replaced with an Alanine, these TFs exhibited diminished DNA binding and transcriptional activity leading to substantial downstream effects including reductions in the expression of key survival genes CA9 and Survivin, decreased mitochondrial function, and lowered cellular proliferation [37]. The profound impact of the C65A mutation on the activity of key transcription factors and tumor progression underscores the therapeutic potential of Ref-1 inhibition and is further validation of our approach to targeting the oncogenic pathways that PDAC cells rely on.

The new Ref-1 inhibitors show substantial promise in our PDAC preclinical models and are capable of effectively targeting the redox function of Ref-1, leading to reduced tumor growth. These inhibitors hold great potential for overcoming drug resistance and improving the efficacy of existing and novel cancer therapies.

### Utilizing Advanced PDAC Models to Improve Drug Development

#### 3D spheroids and organoids in PDAC research

**Figure 2.**
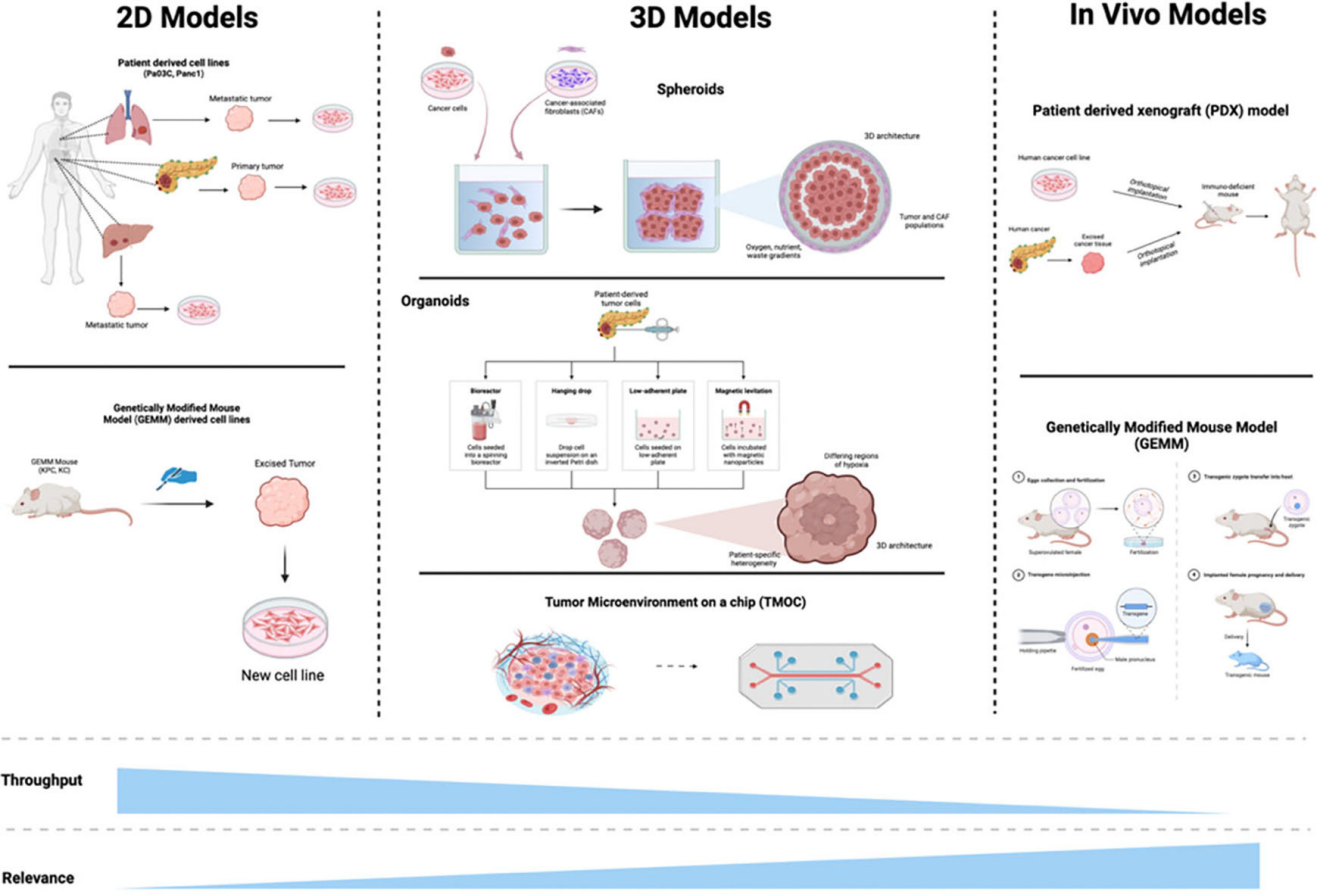
Modeling PDAC: 2D, 3D, and *in vivo* systems. Overview of advanced PDAC models used for research, highlighting 2D, 3D, and *in vivo* systems. 2D Models include patient-derived cell lines, such as PANC-1 and Pa03C, which provide high-throughput but lower physiological relevance, and GEMM-derived cell lines that aid in understanding PDAC-specific mutations. 3D Models offer enhanced structural complexity and include spheroids, which mimic tumor architecture and oxygen gradients, and organoids, which allow for patient-specific studies of drug response [41]. The tumor microenvironment on a chip (TMOC) further simulates PDAC complexity, incorporating aspects like fluid flow and multiple cell types [42]. *In vivo* models provide the highest relevance and include patient-derived xenografts (PDX) and GEMMs for studying tumor behavior in a more physiological context [44]. This spectrum of models balances throughput with relevance, allowing comprehensive exploration of PDAC biology and treatment responses.

To deliver breakthroughs in PDAC treatment, it will not only be necessary to identify and target novel molecular pathways, but it will also be essential to utilize more effective modeling of the complex PDAC environment to ensure that these new therapies are able to be translated clinically [38]. We aim to integrate advanced PDAC modeling techniques that accurately reflect the heterogeneity and microenvironment complexities of PDAC along with our drug development process. This strategy encompasses multiple facets aimed at overcoming the unique challenges of PDAC (Figure 2).

Traditional monolayer (2D) cultures of PDAC cells, although useful, lack the complexity of the tumor microenvironment and cannot replicate the tumor-stromal interactions and the 3D architecture of PDAC. Thus, while we utilize 2D cultures for initial screening, we further employ 3D spheroid and organoid cultures, which represent an advancement over 2D models by allowing cells to grow in a more physiologically relevant manner.

The 3D structure of spheroid models enables the development of oxygen, nutrient, and waste gradients, closely resembling the conditions in solid tumors where cells in the core experience hypoxia and nutrient deprivation. These models recapitulate more realistic cell-matrix interactions which are crucial for processes such as cell migration, invasion, and response to chemotherapy. 3D spheroids can incorporate not only PDAC cells but also CAFs, enabling the study of stromal interactions and their impact on drug efficacy [39]. Organoid cultures better mirror the *in vivo* tumor microenvironment allowing for the preservation of key features such as differentiation gradients and hypoxic regions, which are critical for understanding tumor biology and therapeutic response [40]. Organoids can be derived from patient tumors, maintaining the genetic and phenotypic heterogeneity of the original cancer, which is often lost in 2D cultures [41].

#### Tumor-microenvironment-on-chip (T-MOC) models

We additionally utilize T-MOC models which can effectively simulate the dynamic interactions between tumor cells and their microenvironment [36]. T-MOC models include interstitial channels filled with cell-collagen mixtures that mimic the 3D culture condition, flanked by channels that supply nutrients and drugs. This allows for precise control over the microenvironmental conditions and better simulates the physiological and mechanical properties of tumors [42]. These models also enable the integration of multiple cell types within a controlled environment, allowing for the investigation of complex cell-cell and cell-matrix interactions that drive tumor progression and drug resistance [43]. By providing a more realistic and tunable microenvironment, T-MOC models improve the predictability of therapeutic responses, offering a more robust platform for preclinical testing and the development of targeted therapies.

#### Genetically engineered mouse models and PDX

We, at times, also utilize cell lines derived from genetically engineered mouse models (GEMMs) such as the commonly used KRAS^G12D^; TP53^R172H^; Pdx-1-Cre (KPC) and Kras^G12D^; Pdx1-Cre (KC) models. GEMMs are mice that have been genetically modified to carry mutations that mimic those found in human diseases [44]. Common genetic targets include KRAS, TP53, and SMAD4 which are frequently mutated in human PDAC. These models allow for the study of the initiation, progression, and metastasis of cancers, including the molecular and cellular mechanisms underlying these processes [45].

GEMMs have long been a mainstay of cancer research and have significantly contributed to the understanding of the molecular mechanisms driving PDAC [44,46]. However, while the development of PDAC GEMMs marked a significant advancement in PDAC modeling and research, it has become clear that findings within these models do not always translate directly to human patients. Our use of these cell lines is in keeping with our multifaceted approach wherein we incorporate multiple approaches to modeling and targeting PDAC.

Supplementing our *in vitro* models, we further incorporate *in vivo* orthotopic mouse models within our development pipeline. These provide a more relevant environment for studying human tumor growth and response to treatments by enabling the evaluation of drug efficacy and pharmacodynamics in a living organism. The use of patient-derived xenografts (PDX) further enhances the clinical relevance of these models. PDX models maintain the histopathological and genetic characteristics of the original patient tumors, thereby reflecting the tumor heterogeneity that is a hallmark of PDAC. This approach ensures that our experimental conditions closely mimic the complexity of human cancers, thus improving the predictive power of our preclinical studies.

## Conclusion

The complexity of PDAC presents significant challenges to the development of effective therapies. However, by targeting key molecular pathways such as Ref-1 and its associated transcription factors like STAT3, HIF1α, and NF-κB, we can disrupt crucial survival mechanisms essential for PDAC homeostasis.

The development of second-generation Ref-1 inhibitors that demonstrate enhanced potency and better pharmacokinetic profiles represents a promising advancement in PDAC treatment. Our utilization of advanced preclinical models, including 3D spheroids, organoids, and T-MOC systems, is pivotal in improving the predictability of therapeutic responses. These models provide a more accurate representation of TME and its interactions with therapeutic agents. By simulating the complex conditions within the TME, such as hypoxia and nutrient deprivation, these models allow for the evaluation of drug efficacy in a setting that closely mirrors *in vivo* conditions. The integration of patient-derived xenografts further enhances the clinical relevance of our studies, ensuring that our findings are translatable to patient care.

Our current focus is on refining these preclinical models and further optimizing our Ref-1 inhibitors to enhance their clinical applicability. We aim to integrate these inhibitors into combination therapies, targeting both the tumor cells and their supportive microenvironment. By integrating novel target discovery with robust preclinical models, we aim to streamline the drug development process. Our goal is to reduce the high attrition rates observed in PDAC clinical trials by ensuring that only the most promising therapeutic candidates advance to clinical testing.

## Figures and Tables

**Table 1.** Ref-1 Analogs. List of Ref-1 inhibitors, detailing their structures and mechanisms of action. Each inhibitor binds directly to the Ref-1 protein, specifically targeting and inhibiting its redox activity without interfering with its endonuclease function [36]. Further research is required to fully understand the detailed mechanisms by which these inhibitors modulate Ref-1’s redox function.

Ref-1 Inhibitor	Structure	Mechanism of Action
APX2009	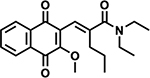	Direct binding to Ref-1 and selective inhibition of redox activity with no impact on endonuclease activity.
APX2014	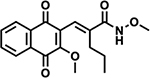	Direct binding to Ref-1 and selective inhibition of redox activity with no impact on endonuclease activity.
APX2044	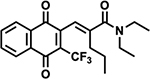	Direct binding to Ref-1 and selective inhibition of redox activity with no impact on endonuclease activity.
APX2051	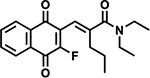	Direct binding to Ref-1 and selective inhibition of redox activity with no impact on endonuclease activity.
APX2053	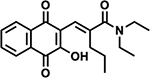	Direct binding to Ref-1 and selective inhibition of redox activity with no impact on endonuclease activity.
